# TNF‐α promotes survival and migration of MSCs under oxidative stress *via* NF‐κB pathway to attenuate intimal hyperplasia in vein grafts

**DOI:** 10.1111/jcmm.13131

**Published:** 2017-03-07

**Authors:** Xiao Bai, Jie Xi, Yanwen Bi, Xin Zhao, Weidong Bing, Xiangbin Meng, Yimin Liu, Zhonglai Zhu, Guangmin Song

**Affiliations:** ^1^ Department of Cardiovascular Surgery Qilu Hospital of Shandong University Jinan Shandong China

**Keywords:** vein graft, intimal hyperplasia, oxidative stress, MSCs, NF‐κB, TNF‐α

## Abstract

The oxidative stress caused by endothelial injury is involved in intimal hyperplasia (IH) in vein grafts. Mesenchymal stem cells (MSCs) can home to injured intima and promote endothelial repair. However, MSC apoptosis is increased accompanied by decreased functional activity under oxidative stress. Thus, we investigate whether tumour necrosis factor‐α (TNF‐α) can promote the survival and activity of MSCs under oxidative stress to reduce IH more effectively, and establish what role the NF‐κB pathway plays in this. In this study, we preconditioned MSCs with TNF‐α (^TNF^ ^‐α‐PC^MSCs) for 24 hrs and measured the activation of the IKK/NF‐κB pathway. EdU and transwell assays were performed to assess proliferation and migration of ^TNF^ ^‐α‐PC^MSCs. Apoptosis and migration of ^TNF^ ^‐α‐^ ^PC^MSCs were evaluated in conditions of oxidative stress by analysis of the expression of Bcl‐2 and CXCR4 proteins. ^TNF^ ^‐α‐^ ^PC^MSCs were transplanted into a vein graft model, so that cell homing could be tracked, and endothelial apoptosis and IH of vein grafts were measured. The results demonstrated that TNF‐α promotes proliferation and migration of MSCs. Furthermore, survival and migration of ^TNF^ ^‐α‐^ ^PC^MSCs under oxidative stress were both enhanced. A greater number of MSCs migrated to the intima of vein grafts after preconditioning with TNF‐α, and the formation of neointima was significantly reduced. These effects could be partially abolished by IKK XII (NF‐κB inhibitor). All these results indicate that preconditioning with TNF‐α can promote survival and migration of MSCs under oxidative stress *via* the NF‐κB pathway and thus attenuate IH of vein grafts.

## Introduction

Autologous vein grafts are commonly used for myocardial revascularization around the world. Disappointingly, several lines of evidence suggest that approximately 40–50% of these vein grafts ultimately result in stenosis and even occlusion 1, 2, 3. The primary cause of stenosis of vein grafts is oxidative stress caused by injury of the endothelium 4, 5.

MSCs are mobilized to peripheral blood from the bone marrow and then home to injured vascular intima to promote endothelial repair 6, 7. Because of their many advantages, such as ease of isolation and culture, their directional migration and lack of immune rejection, MSCs are considered to have great therapeutic potential for many diseases 8, 9. Injection of MSCs has been utilized to alleviate atherosclerosis, myocardial ischaemia and vascular balloon injury 10, 11, 12. Experiments have shown that oxidative stress exists in vascular intima and the blood circulation following both vein grafting and vascular injury, after a short period of time and lasting for several weeks 4, 5. Furthermore, recent studies have demonstrated that excessive and/or persistent stimulation of oxidative stress can reduce the functions of MSCs and increase apoptosis 13, 14. As a consequence, the therapeutic effect of the transplantation of MSCs can be seriously reduced.

It was reported that up‐regulation of the nuclear factor of kappa B (NF‐κB) pathway can promote anti‐apoptosis of cells under oxidative stress 15. Bcl‐2 protein has been thought of as an anti‐apoptotic protein 16, 17. Therefore, we evaluated the anti‐apoptotic effect of NF‐κB pathway activation in MSCs under oxidative stress, with Bcl‐2 as an indicator. MSCs also secrete vascular endothelial growth factor (VEGF) and hepatocyte growth factor (HGF), which can promote endothelial growth and reduce apoptosis 15, 18. Paracrine may be one way in which MSCs reduce endothelial apoptosis after vein grafting.

TNF‐α is an important pro‐inflammatory cytokine. The NF‐κB pathway can be activated by a large variety of factors, including cytokines and stress stimuli 19, 20, 21. In this study, we attempted to activate the NF‐κB pathway by stimulation with TNF‐α, so that specific molecular mechanisms could be explored.

It is widely accepted that the SDF‐1α/CXCR4 axis has a role in mediating the migration of stem cells or tumour cells, one which can be inhibited by the CXCR4 antagonist, AMD3100 22, 23, 24. Besides, the CXCR4 promoter region has been shown to contain NF‐κB response elements 25. Therefore, we wish to determine whether overexpression of CXCR4 stimulated by TNF‐α *via* the NF‐κB pathway could enhance the migration of MSCs under oxidative stress.

In this study, we investigated the effect of the TNF‐α on a variety of activities of MSCs, such as survival, migration and secretion. The findings may shed light on a possible role of the NF‐κB pathway in prevention of IH in vein grafts with the help of MSCs.

## Materials and methods

### Isolation, culture and labelling of MSCs

MSCs were extracted from the femurs of young Wistar rats (male, 2 weeks) under anaesthesia with 1% pentobarbital sodium (40 mg/kg). The culture medium was ɑ‐MEM (HyClone, Logan, UT, USA) with 10% foetal bovine serum (FBS) (Clarkbio, Richmond, VA, USA). The bone marrow was flushed with culture medium and then cultured at 37°C in 5% CO_2_. Cell purification was achieved by washing with phosphate‐buffered saline (PBS) and changing the culture medium after 48 hrs. Cells at passage 3 to 4 were used in the experiments. Before transplantation, cells were labelled with chloromethylbenzamido dialkylcarbocyanine (CM‐Dil) for cell tracking *in vivo* according to the manufacturer's instruction (Invitrogen, Carlsbad, CA, USA).

### Chemical treatment of MSCs

MSCs were preconditioned with TNF‐α (50 ng/ml) (BioLegend, San Diego, CA, USA) for 24 hrs. Treatment with IKK XII (NF‐κB inhibitor, 5 μM) (Merck, Kenilworth, NJ, USA) and/or SB203580 (p38 MAPK inhibitor, 5 μM) (Selleck, Houston, TX, USA) was carried out prior to TNF‐α treatment for 30 min. to determine the role of NF‐κB or p38 MAPK pathway. To investigate the survival and migration of TNF‐α‐ and/or IKK XII‐preconditioned MSCs under oxidative stress, medium was exchanged after 24 hrs of chemical pre‐treatment, and then, MSCs were treated with H_2_O_2_ (200 μM) for 12 hrs.

### Vein grafting models

Adult male Wistar rats weighing 250–300 g were used in this experiment. The left external jugular vein (LEJV) was inserted into the left common carotid artery (LCCA) in the same animal. Vein grafting was performed with the cuff technique 26. Heparin (100 U/100 g) was used before and after grafting. CM‐Dil‐labelled cells (10^8^/ml × 0.2 ml) were transplanted through the caudal vein after grafting. Experimental rats were randomly divided into five groups: (*i*) sham group (skin incision without vein grafting), *n* = 12; (*ii*) untreated group (vein grafting without MSC transplantation), *n* = 12; (*iii*) ^Non‐PC^MSC group (vein grafting and treatment with non‐preconditioned MSCs), *n* = 24; (*iv*) ^TNF‐α‐PC^ MSC group (vein grafting and treatment with TNF‐α‐preconditioned MSCs), *n* = 24; and (*v*) ^IKKXII+TNF‐α‐PC^MSC group (vein grafting and treatment with IKK XII +TNF‐α‐preconditioned MSCs), *n* = 24.

In each MSC transplantation group, 12 rats were humanely killed 1 week after operation for evaluation of endothelial apoptosis using a TUNEL assay, and MSC homing using fluorescence microscopy. All remaining rats were killed 4 weeks after the operation. Van Gieson staining was performed to evaluate IH. All of the study protocols were approved by the Animal Care Committee of Qilu Hospital of Shandong University. All animals were cared according to the Guide for the Care and Use of Laboratory Animals.

### Cell‐surface phenotype analysis

Flow cytometry was used to identify the phenotypes of the cultured MSCs. MSCs at passage 3 were incubated with anti‐CD29, anti‐CD44, anti‐CD45, anti‐CD90 (eBioscience, San Diego, CA, USA) or anti‐CD34 (Santa CruzBiotechnology, Santa Cruz, CA, USA). Antibodies of the corresponding isotype served as the negative control. To detect CXCR4 expression on the cellular surface, cells were stained with mouse anti‐rat CXCR4 antibody (Sigma, St. Louis, MO, USA) or the isotype control. The secondary antibody was donkey anti‐mouse IgG PE (Proteintech, Chicago, IL, USA).

### Western blot

Protein samples were loaded onto polyacrylamide gels (8–10%) for electrophoresis. The protein was then transferred onto a polyvinylidene fluoride (PVDF) membrane. Each membrane was blocked in tris‐buffered saline with Tween 20 (TBST) containing 5% non‐fat dry milk for 1 hr at room temperature and then incubated at 4°C overnight with one of the following primary antibodies: anti‐IKK‐α (1:1000, Abcam, Cambridge, MA, USA); anti‐IKK‐β (1:1000; Abcam); anti‐p‐IKK‐α/β (Ser176/180) (1:1000, CST, Danvers, MA, USA); anti‐NF‐κB‐p65 (1:1000; Abcam); anti‐p‐NF‐κB‐p65 (S536) (1:1000; Abcam); anti‐p38MAPK (1:1000; CST); anti‐p‐p38MAPK (Thr180/Tyr182) (1:1000; CST); anti‐CXCR4 (1:1000; Sigma‐Aldrich); anti‐Bcl‐2 (1:200; CST); or anti‐GAPDH (1:1000; Proteintech). This was followed by incubation in peroxidase‐conjugated AffiniPure goat anti‐rabbit or mouse IgG (H+L) (1:5000, MultiSciences, Hangzhou, Zhejiang, China) for 1 hr at room temperature. Immunoreactivity was detected by chemiluminescence.

### Analysis of cell cycle and apoptosis

Cell cycle was detected using Cycletest Plus DNA Reagent Kit (BD Biosciences, San Jose, CA, USA) according to the manufacturer's instruction. To quantify the apoptosis of the MSCs, flow cytometry was performed with an annexin V‐FITC/PI apoptosis detection kit (BD Biosciences).

### Cell viability assay

Cell viability was assessed using a Cell Counting Kit‐8 (CCK‐8) according to the manufacturer's protocol (Dojindo, Kumamoto, Japan). MSCs were seeded onto 96‐well plates at a density of 6 × 10^3^ cells per well. Following overnight incubation, cells were treated with different reagents for 12–24 hrs, and 10% CCK‐8 solution was then added to each well. The absorbance of the supernatant was measured at a wavelength of 450 nm after the cells were incubated for 2 hrs at 37°C.

### EdU cell proliferation assay

Cell proliferation was assessed using an EdU DNA Cell Proliferation Kit according to the manufacturer's protocol (Ribobio, Guangzhou, China). The nuclei of cells in proliferation state showed red. The proliferation rate = the number of cells in proliferation state (red)/the number of total cells (purple).

### Immunofluorescence microscopy

Preconditioned MSCs cultured on glass slides were fixed with 4% paraformaldehyde for 20 min. and permeabilized with 0.5% Triton X‐100 for 20 min. Cellular proteins were detected with primary antibodies against p‐NF‐κB‐p65 and CXCR4. FITC‐conjugated goat anti‐rabbit and PE‐conjugated donkey anti‐mouse secondary antibodies were used. Nuclei were stained using 4′, 6′‐diamidino‐2‐phenylindole (DAPI) (Sigma‐Aldrich). Coverslips were mounted with FluorSave Reagent (Solarbio, Beijing, China). Images were acquired using fluorescence microscopy.

### Transwell migration assay

Migration assay was performed with a transwell (CorningInc., Corning, NY, USA) containing a polycarbonate membrane filter (8 μm pore size). 200 μl preconditioned MSCs (1.5 × 10^5^/ml) was seeded in the upper chamber and 600 μl complete medium with SDF‐1a (100 ng/ml, Proteintech, IL, USA) was placed into the lower well to induce cell migration. Following 12‐hrs incubation, cells on the upper side of the membrane were removed using cotton swabs. Cells on the bottom surface of the membrane were stained with 0.1% crystal violet and counted in five randomly selected microscopic fields (×200).

### ELISA for MDA, VEGF and HGF

Rat blood samples were obtained 1 day and 1 week after vein grafting, and serum was isolated from each tube after centrifugation at 500 × *g* for 15 min. Methane dicarboxylic aldehyde (MDA) concentrations in the serum were measured to assess the level of oxidative stress using an ELISA kit (ExCellBio, Shanghai, China). MSCs were preconditioned for 24 hrs, and the culture medium of each well was collected. VEGF and HGF protein concentrations in each well were quantified using an enzyme‐linked immunosorbent assay (ELISA) kit (ExCellBio) according to the manufacturer's instructions.

### TUNEL assay

The apoptosis of endothelial cells in vein graft was observed using a TUNEL Andy Fluor™ 488 Apoptosis Detection Kit (GeneCopoeia, Rockville, MD, USA). Endothelial cells were labelled according to the manufacturer's instructions. Stained sections were observed using fluorescent microscopy. We counted the nuclei of vascular endothelium (luminal border of the blood vessel). The nuclei of apoptotic cells showed green. The apoptosis rate = the number of apoptotic cells (green)/the number of total cells (purple).

### Van Gieson stain

Vein grafts were fixed in 4% paraformaldehyde and then embedded in paraffin. The paraffin specimens were sectioned into 4‐μm‐thick slices. Briefly, sections were prepared by dewaxing, staining and then dehydration. The morphology of the vascular intima was observed by light microscopy.

### Statistical analysis

All values were presented as mean ± S.D. Data were analysed using one‐way analysis of variance (anova) followed by Dunnett's multiple comparison tests. Values of *P* < 0.05 were considered statistically significant. All analyses were performed with SPSS19.0 software (SPSS Inc., Chicago, IL, USA).

## Results

### Characterization of MSCs *in vitro* culture

**Figure 1:**
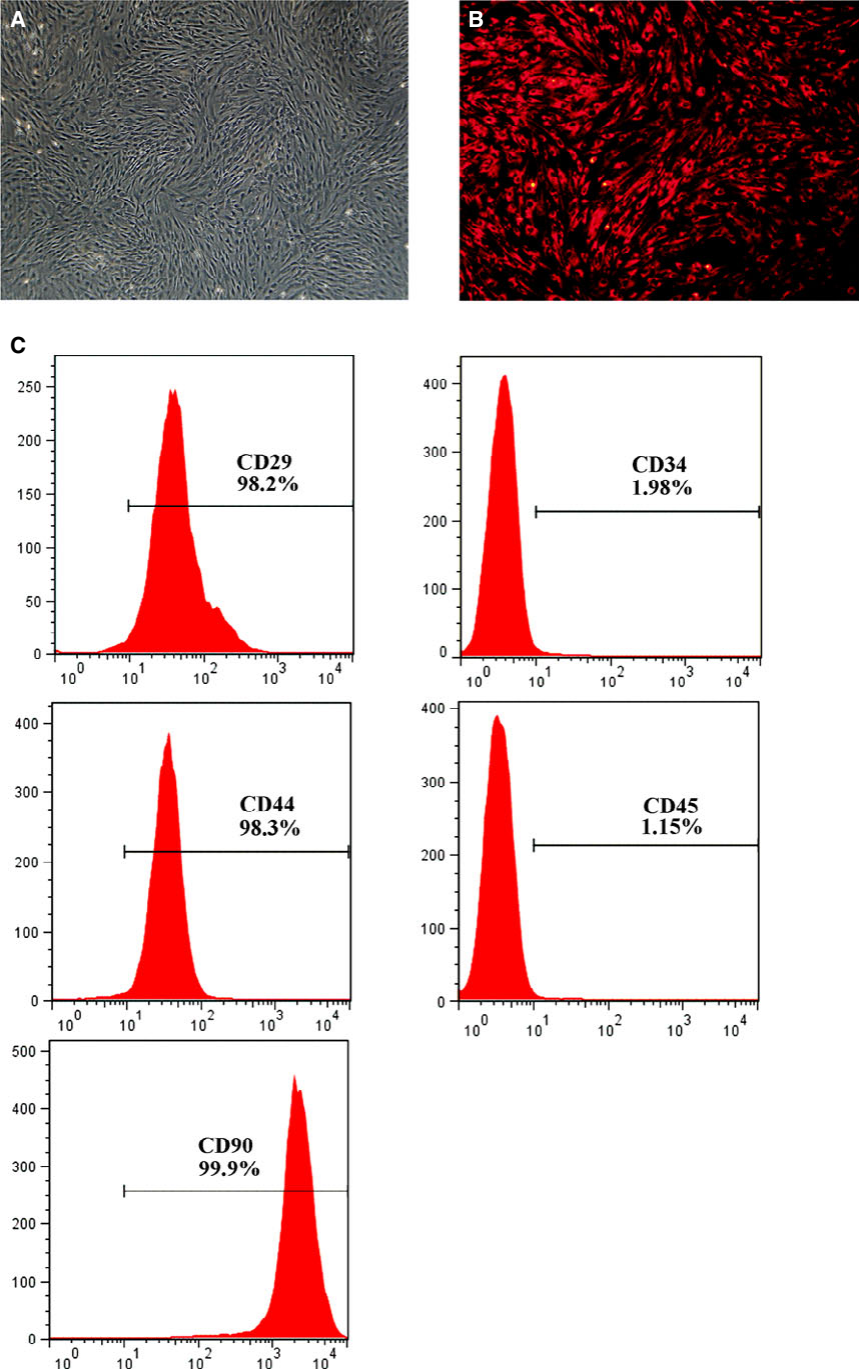
Characterization of MSCs. (**A**) Cultured MSCs at passage 3 presented a flattened fibroblast‐like morphology. (**B**) CM‐Dil‐labelled MSCs emitting red fluorescence under excitation. (**C**) Flow cytometric analysis of cultured cells with CD29, CD34, CD44, CD45 and CD90 antibodies. Results validated the mesenchymal origin of the cell population used in our study.

The MSCs cultured at passage 3 *in vitro* presented a homogeneous fibroblast‐like form (Fig. 1A). The majority of MSCs labelled with CM‐Dil showed red fluorescence under fluorescence microscopy (Fig. 1B). Flow cytometry demonstrated that the cells were uniformly negative for the hematopoietic markers CD34 and CD45, and positive for the stem cell antigens CD29, CD44 and CD90 (Fig. 1C). Thus, the phenotype of the cell population used in our study was consistent with that of MSCs.

### TNF‐α activates NF‐κB pathway by increasing phosphorylation of IKK‐α/β

**Figure 2:**
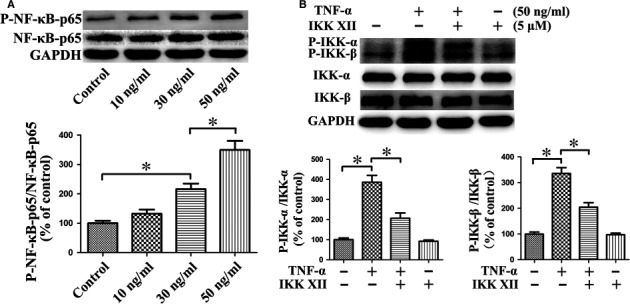
TNF‐α activates NF‐κB pathway by increasing phosphorylation of IKK‐α/β. (**A**) Phosphorylation level of NF‐κB‐p65 was elevated after stimulation by TNF‐α, whereas the level of NF‐κB‐p65 was not significantly changed. The activation effect was dose dependent. *P < 0.05. (**B**) IKK‐α and IKK‐β are pivotal upstream regulatory proteins of the NF‐κB pathway. TNF‐α increased phosphorylation of IKK‐α and IKK‐β, with activation effectively antagonized by IKK XII. *P < 0.05.

TNF‐α mainly caused an increase in the phosphorylation of NF‐κB‐p65, whereas the level of NF‐κB‐p65 did not change significantly. TNF‐α activated the NF‐κB pathway, with activation gradually increasing in proportion to the increase in dose (Fig. 2A). IKK‐α and IKK‐β are pivotal upstream regulatory proteins of the NF‐κB pathway. The degree of phosphorylation of IKK‐α and IKK‐β increased significantly following preconditioning with TNF‐α. The phosphorylation could be effectively antagonized by the IKK‐α/β inhibitor IKK XII (Fig. 2B). These results confirmed that TNF‐α could activate the NF‐κB pathway by increasing the phosphorylation of IKK‐α/β.

### NF‐κB pathway plays the leading role in regulating cell viability and proliferation

**Figure 3:**
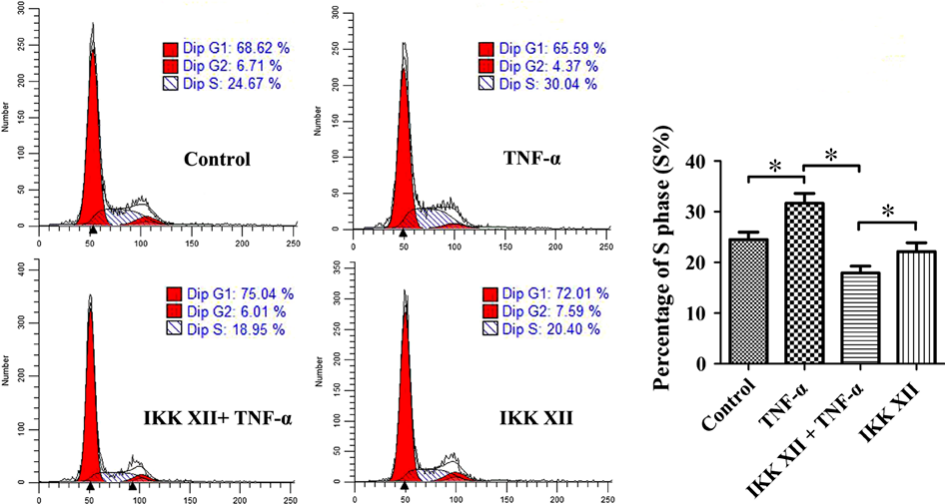
Effects of TNF‐α and/or IKK XII on the cell cycle of MSCs. The percentage of cells in S phase could reflect cell proliferation in some extent. The percentage of cells in S phase was increased by TNF‐α preconditioning and inhibited by IKK XII. *P < 0.05.

**Figure 4:**
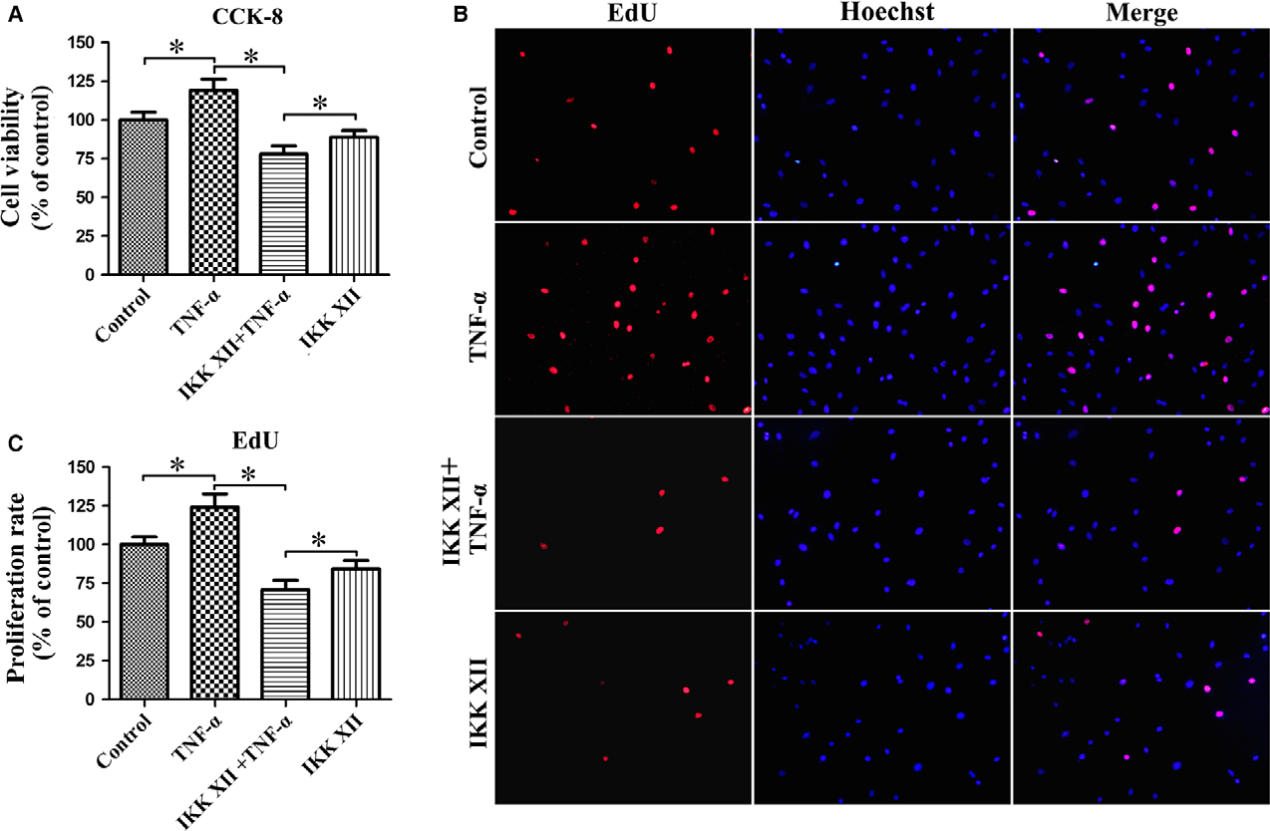
TNF‐α promotes cell viability and proliferation. (**A**) TNF‐α increased total cell viability, which was reduced by IKK XII antagonizing the NF‐κB pathway. Total cell viability of IKK XII+TNF‐α group was reduced compared with IKK XII group. *P < 0.05. (**B**) The EdU assay results mainly attributed the increase in total cell viability to the change of cell number rather than cell state. Representative pictures were taken at a magnification of 200×. (**C**) TNF‐α promoted cell proliferation, which was reduced by IKK XII antagonizing the NF‐κB pathway. *P < 0.05.

**Figure 5:**
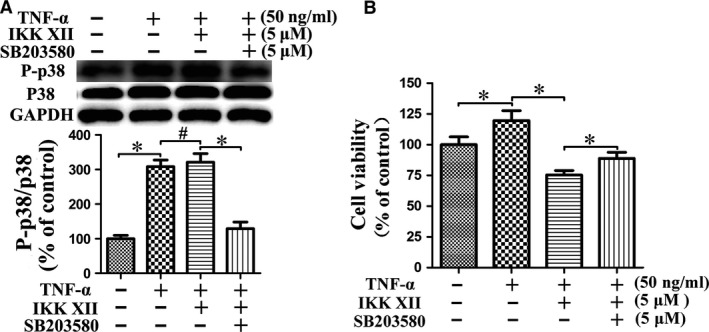
NF‐κB pathway plays the leading role in regulating cell viability. (**A**) TNF‐α also activated the p38‐MAPK pathway, and the activation can be antagonized by SB203580. (**B**) Following IKK XII antagonizing TNF‐α‐activated NF‐κB pathway, cell viability was raised by SB203580 blocking the P38‐MAPK pathway. *P < 0.05; ^#^ *P* > 0.05.

The cell cycle assay showed that the percentage of cells in S phase was significantly increased in the TNF‐α group, the effect of which could effectively be reversed using IKK XII (Fig. 3). We speculated that the NF‐κB pathway promoted cell proliferation. The CCK‐8 assay demonstrated that TNF‐α raised total cell viability significantly (Fig. 4A). The EdU assay suggested that TNF‐α promoted cell proliferation significantly, and the increased total cell activity revealed by CCK‐8 assay was mainly contributed by the change of cell number rather than cell state (Fig. 4B and C). However, we observed that cell viability and proliferation of the IKK XII+TNF‐α group were lower than the IKK XII group. Further studies suggested that TNF‐α also activated the p38‐MAPK pathway, which was related to cell apoptosis (Fig. 5A). We speculated that when the activated NF‐κB pathway was blocked by IKK XII, the pro‐apoptotic effect of the p38‐MAPK pathway was expressed. The CCK‐8 results demonstrated that cell viability then increased, after the p38‐MAPK pathway was additionally antagonized by SB203580 (Fig. 5B). Thus, we confirm that the NF‐κB pathway plays the leading role in regulating cell viability and proliferation.

### TNF‐α promotes CXCR4 expression and enhances migration function in MSCs *via* NF‐κB pathway

**Figure 6:**
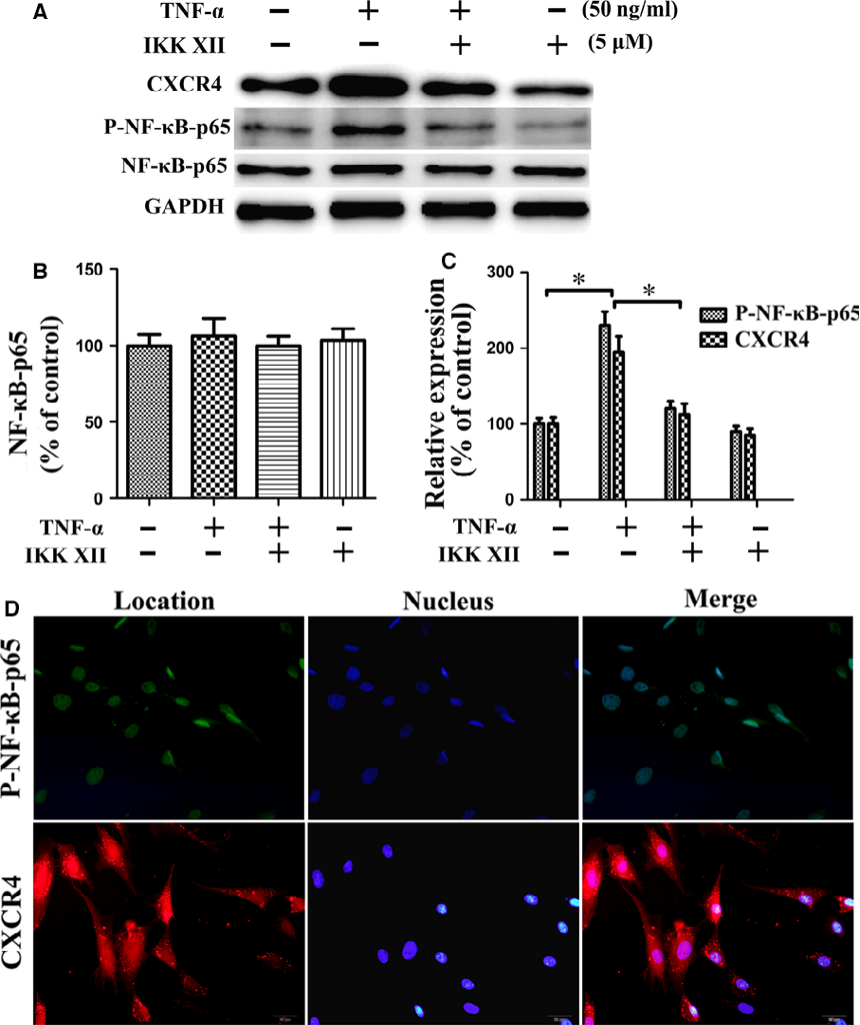
TNF‐α promotes CXCR4 expression in MSCs *via* the NF‐κB pathway. (**A**) Expression of p‐NF‐κB‐p65 and CXCR4. (**B**) Expression of NF‐κB‐p65 showed no significant change. (**C**) TNF‐α increased p‐NF‐κB‐p65 and CXCR4 expression. Pre‐treatment with IKK XII abolished the overexpression. The expression levels of p‐NF‐κB‐p65 and CXCR4 had almost the same change trend. *P < 0.05. (**D**) Immunofluorescence microscopy shows that p‐NF‐κB‐p65 (green) is normally present in the nuclei. CXCR4 (red) is observed mainly in the cytoplasm.

**Figure 7:**
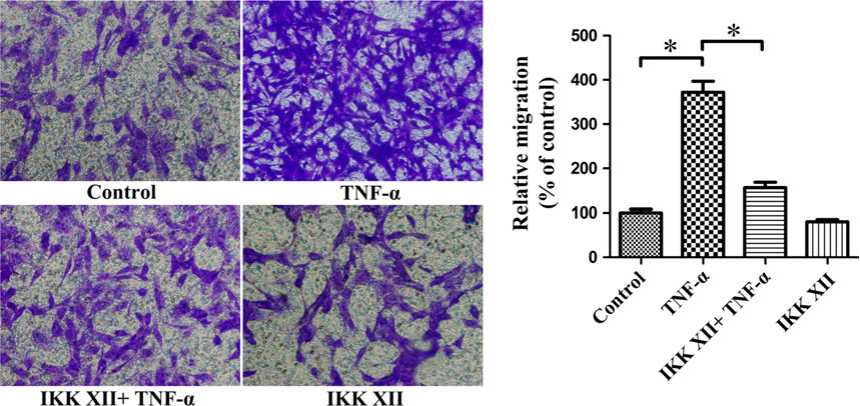
TNF‐α improves migration of MSCs towards SDF‐1α *via* NF‐κB pathway. IKK XII reduces the improvement of migration caused by TNF‐α. Representative pictures were taken at a magnification of 200×. *P < 0.05.

**Figure 8:**
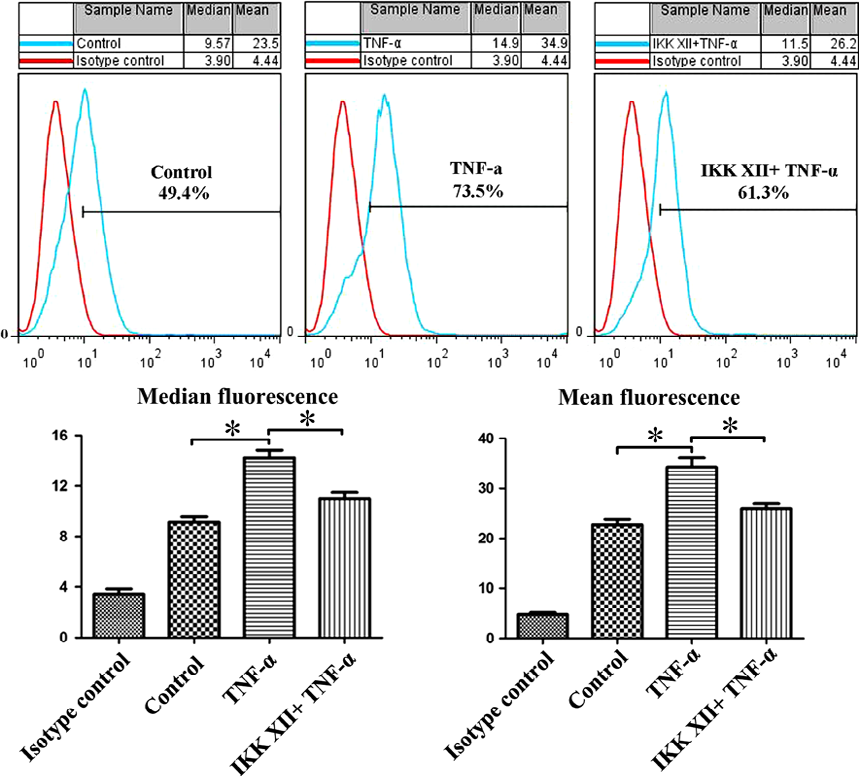
CXCR4 expression on the surface of MSCs was significantly increased in the TNF‐α group. IKK XII could reduce the overexpression of CXCR4 caused by TNF‐α. *P < 0.05.

To evaluate the role of the NF‐κB pathway in regulating the migration of MSCs towards SDF‐1α, the expressions of p‐NF‐κB‐p65/NF‐κB‐p65 and CXCR4 were examined (Fig. 6A). TNF‐α treatment increased p‐NF‐κB‐p65 expression, whereas the NF‐κB‐p65 levels were not significantly altered (Fig. 6B). The expression of CXCR4 was increased in the TNF‐α group. Furthermore, pre‐treatment with IKK XII abolished the overexpression of CXCR4 caused by TNF‐α. Therefore, CXCR4 expression may be related to p‐NF‐κB‐p65 levels (Fig. 6C). P‐NF‐κB‐p65 is normally present in the nuclei, playing a regulatory role. CXCR4 is observed mainly in the cytoplasm, exerting a specific influence (Fig. 6D). We considered that TNF‐α may promote CXCR4 expression and enhance the migration function in MSCs *via* the NF‐κB pathway. Moreover, the transwell migration assay results also supported this finding (Fig. 7). Cell‐surface phenotype analysis indicated that CXCR4 expressed on surface of MSCs increased following treatment with TNF‐α (Fig. 8). This may be the direct cause of enhanced migration of ^TNF‐α‐PC^ MSCs.

### TNF‐α reduces apoptosis and improves migration of MSCs under H_2_O_2_‐induced oxidative stress *via* NF‐κB pathway

**Figure 9:**
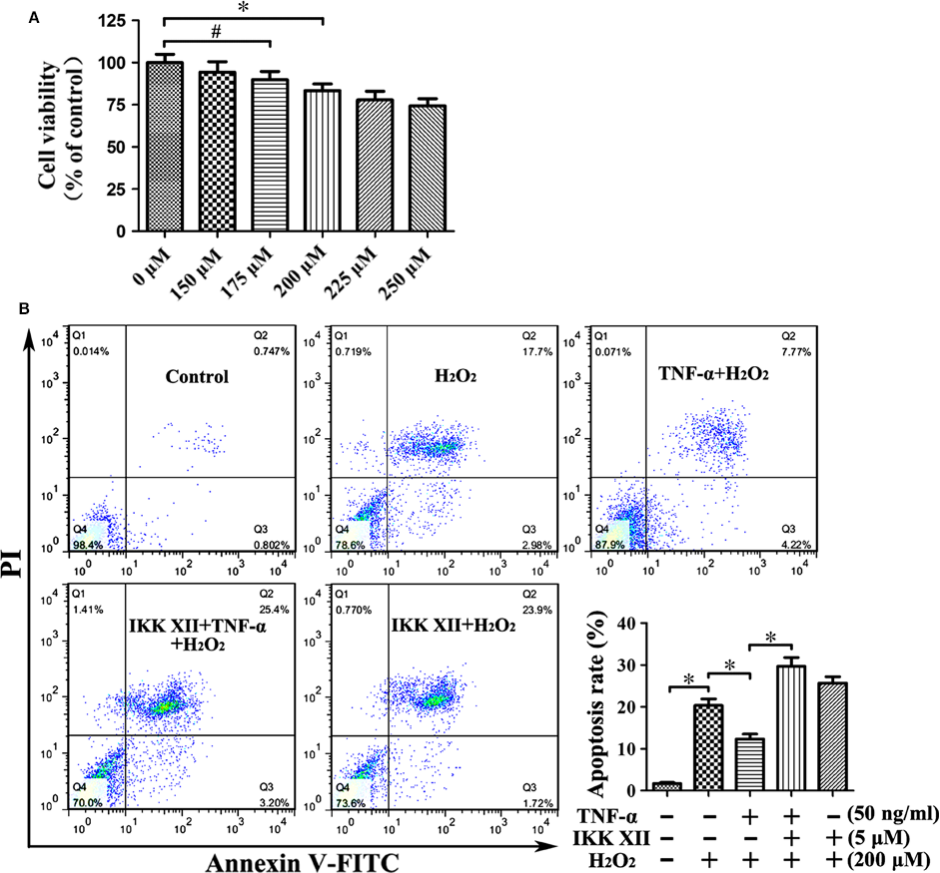
The protective effect of TNF‐α on H_2_O_2_‐induced apoptosis. (**A**) Treatment with 200 μM H_2_O_2_ for 12 hrs reduced viability significantly. *P < 0.05; ^#^ *P* > 0.05. (**B**) 200 μM H_2_O_2_ treatment led to a significant degree of apoptosis. Preconditioning with TNF‐α significantly reduced the apoptosis caused by oxidative stress. This anti‐apoptotic effect was reversed by IKK XII. *P < 0.05.

**Figure 10:**
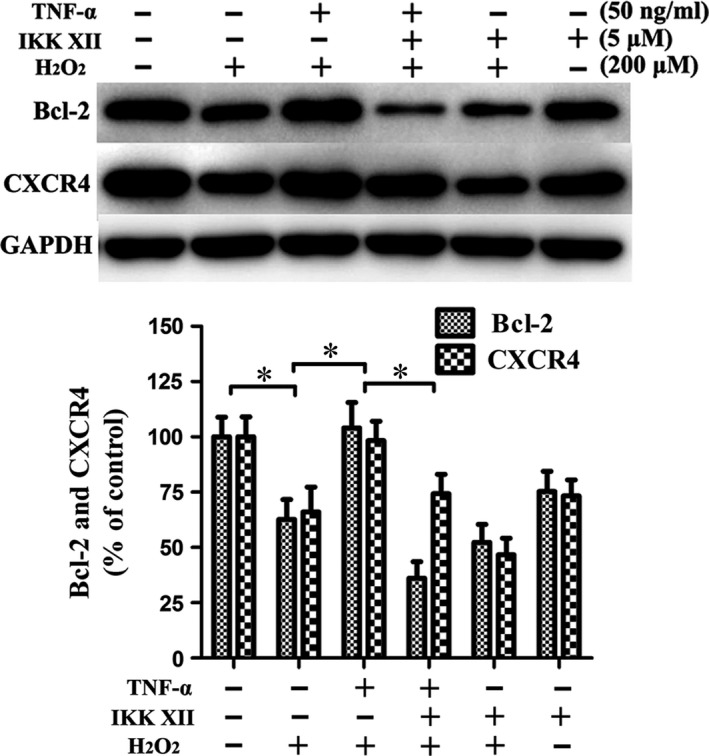
Following treatment with 200 μM H_2_O_2_, the expression levels of Bcl‐2 and CXCR4 were markedly decreased. Preconditioning with TNF‐α resulted in the overexpression of Bcl‐2 and CXCR4 under oxidative stress, and these effects can be abolished by IKK XII. *P < 0.05.

Following treatment with 200 μM H_2_O_2_ for 12 hrs, the viability of MSCs was markedly decreased (Fig. 9A). Flow cytometry demonstrated that H_2_O_2_‐induced oxidative stress gave rise to significant apoptosis of MSCs. Preconditioning with TNF‐α significantly reduced apoptosis caused by oxidative stress. The anti‐apoptosis effect was reversed by IKK XII antagonism of the NF‐κB pathway (Fig. 9B). The anti‐apoptotic effect may be related to up‐regulation of Bcl‐2. Results suggested that when TNF‐α had up‐regulated Bcl‐2, the apoptosis due to oxidative stress was markedly reduced. In addition, TNF‐α elevated the expression of CXCR4, while oxidative stress down‐regulated CXCR4, suggesting migration was increased under oxidative stress after TNF‐α preconditioning. Moreover, the overexpression of Bcl‐2 and CXCR4 caused by TNF‐α could be abolished by IKK XII (Fig. 10). These results confirmed that TNF‐α can reduce apoptosis and increase migration of MSCs under oxidative stress *via* the NF‐κB pathway.

### The protective effect of ^TNF‐α‐PC^MSCs on endothelial cells from oxidative stress injury after vein grafting

**Figure 11:**
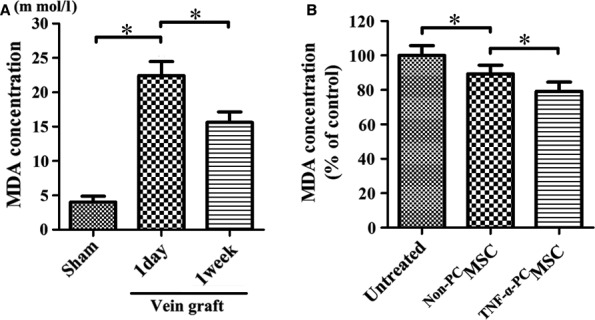
^TNF^ ^‐α‐^ ^PC^MSCs treatment could reduce oxidative stress in blood after vein grafting. (**A**) The serum level of MDA in rat was significantly increased following vein grafting. There was high level of oxidative stress caused by vein grafting in the blood circulation. (**B**) MDA concentration was significantly reduced by treatment of ^TNF^ ^‐α‐^ ^PC^MSC 1 week after vein grafting. *P < 0.05.

**Figure 12:**
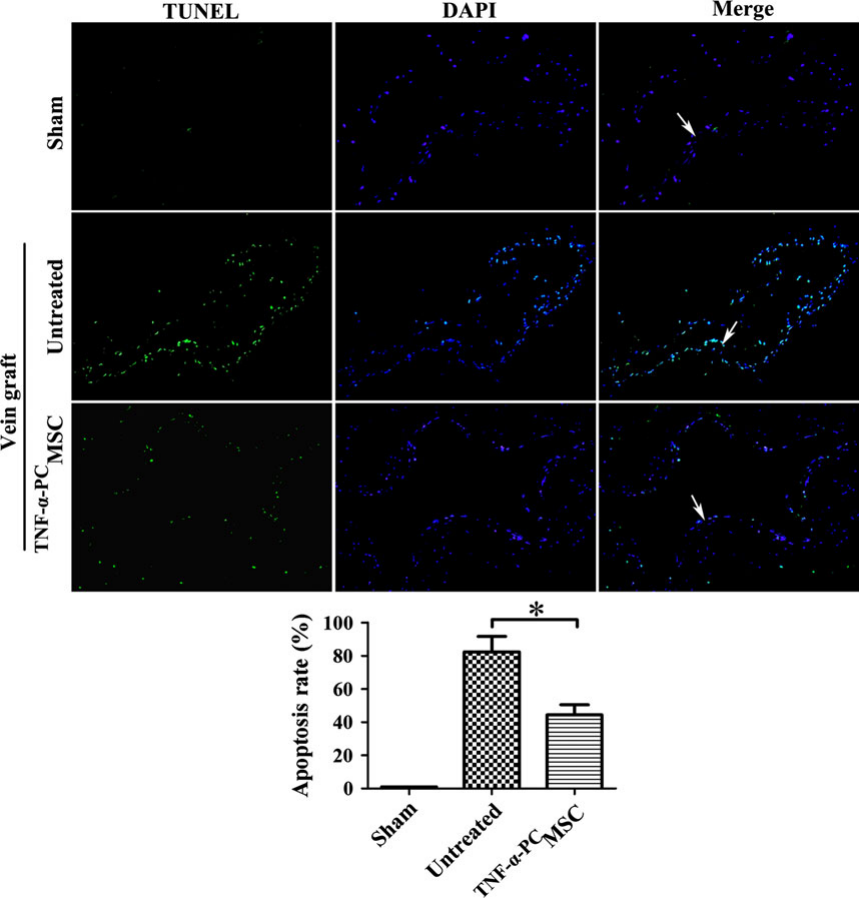
^TNF^ ^‐α‐^ ^PC^MSCs could protect endothelial cells from oxidative stress injury after vein grafting. Mostly endothelial cells were apoptotic 1 week after operation in the untreated group. ^TNF^ ^‐α‐^ ^PC^MSCs treatment reduced the endothelial cell apoptosis significantly. The arrows indicate vascular endothelium. Representative pictures were taken at a magnification of 100×. *P < 0.05.

**Figure 13:**
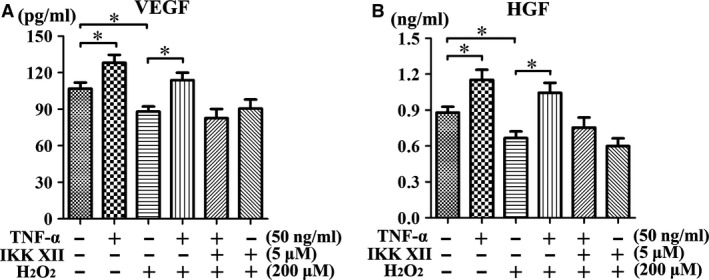
Secretion of pro‐survival factors in ^Non‐^ ^PC^MSCs was diminished under oxidative stress. The secretion of both (**A**) VEGF and (**B**) HGF was significantly elevated in ^TNF^ ^‐α‐^ ^PC^MSCs with or without H_2_O_2_. *P < 0.05.

MDA is a commonly used indicator of membrane lipid peroxidation, reflecting the level of oxidative stress in blood or tissue. An ELISA indicated that the serum level of MDA was significantly increased following vein grafting, giving positive confirmation that oxidative stress resulted from vein grafting in the rat blood circulation (Fig. 11A). Moreover, MDA concentration was significantly reduced by treatment of ^TNF‐α‐PC^MSC 1 week after vein grafting (Fig. 11B). Many researchers hold the view that endothelial apoptosis is the main cause of IH. The TUNEL assay revealed that endothelial cells become apoptotic after vein grafting, but ^TNF‐α‐PC^MSC treatment could significantly delay the process (Fig. 12). The protective effect of ^TNF‐α‐PC^MSCs on endothelial cells may be related to the secretion of pro‐survival factors, such as VEGF and HGF. Indeed, secretions of VEGF and HGF were both significantly elevated in ^TNF‐α‐PC^MSCs with or without H_2_O_2_ (Fig. 13). Experiments confirmed that ^TNF‐α‐PC^MSCs could better protect endothelial cells from oxidative stress injury after vein grafting.

### ^TNF‐α‐PC^MSCs can more efficiently migrate to the damaged vascular intima and attenuate IH in vein grafts

**Figure 14:**
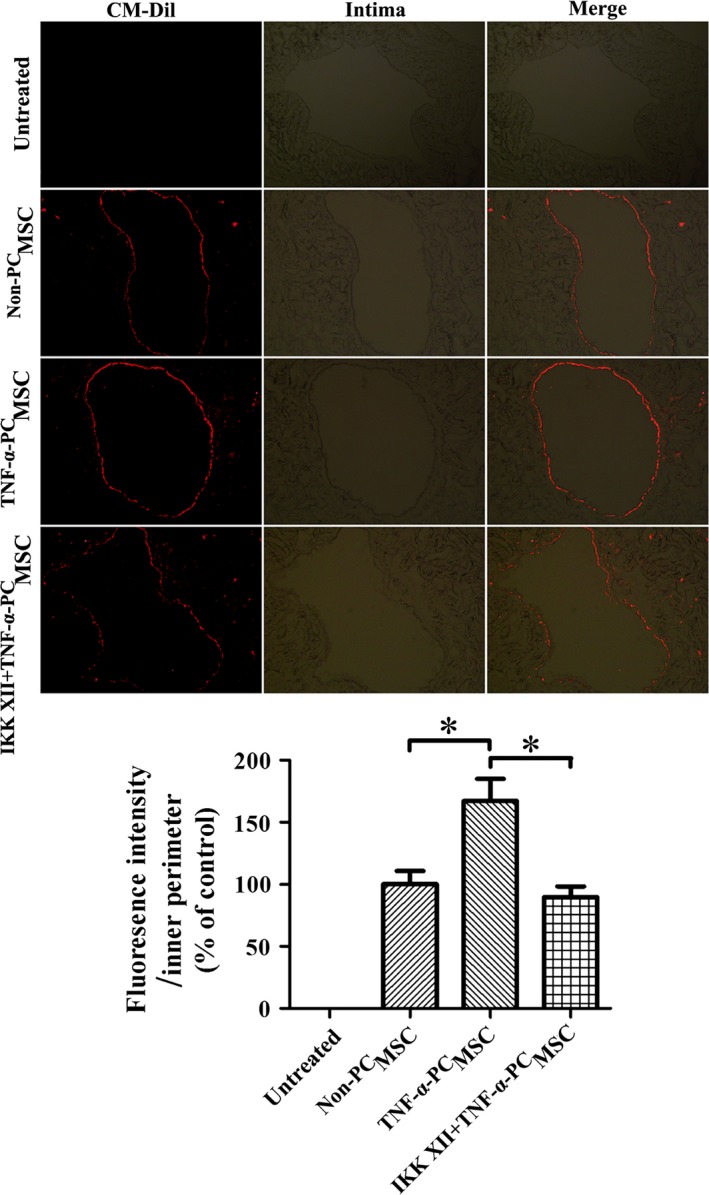
Migration of preconditioned MSCs to the damaged vascular intima 1 week after the operation. More CM‐Dil‐labelled MSCs migrated to the intima following TNF‐α preconditioning. This effect was diminished by pre‐treatment with IKK XII. Representative pictures were taken at a magnification of 200×. *P < 0.05.

**Figure 15:**
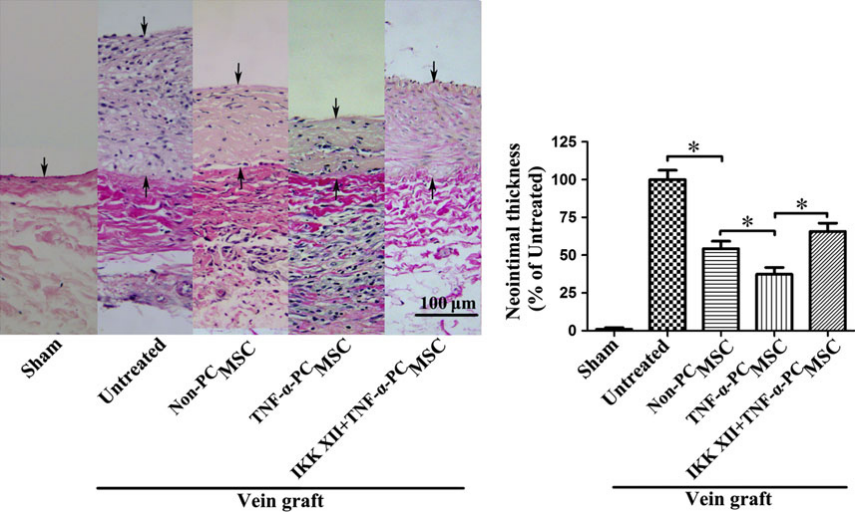
Van Gieson stain analysis of neointima formation in vein grafts 4 weeks after operation. The arrows indicate vascular intima. Formation of neointima was significantly reduced in the ^TNF^ ^‐α‐^ ^PC^MSC group compared with the ^Non‐^ ^PC^MSC group. Thus, ^TNF^ ^‐α‐^ ^PC^MSCs can more efficiently attenuate IH in vein grafts. *P < 0.05.

Homing of MSCs was observed in the vein grafting models. Migration of ^TNF‐α‐PC^MSCs to the intima of the vein graft was significantly increased compared with ^Non‐PC^MSCs, an effect that was reversed by pre‐treatment with IKK XII (Fig. 14). Van Gieson staining demonstrated that the neointima of the ^TNF‐α‐PC^MSC group was also thinner than that of the ^Non‐PC^MSC group, IH in the vein graft thus significantly reduced (Fig. 15). These data suggest that ^TNF‐α‐PC^MSCs can more efficiently migrate to the damaged vascular intima. The effect may derive from the synthetic action of enhanced anti‐apoptosis and ameliorative migration, thus attenuating IH in vein grafts.

## Discussion

Intimal injury is usually considered as the start event for the development of IH. Endothelial cells (ECs) serve as an important barrier, protecting against monocyte adhesion and the proliferation of vascular smooth muscle cells (VSMCs). The apoptosis of ECs will destroy the vascular barrier and expose VSMCs to the blood flow directly, leading to the adhesion and activation of inflammatory cells 1. Activated inflammatory cells then generate excessive reactive oxygen species (ROS), including H_2_O_2_ and superoxide anions. These ROS not only lead to cell apoptosis but also induce the production of inflammatory factors, which may gather more inflammatory cells at injury sites 14. This vicious cycle results in the production of massive ROS, which can constantly aggravate oxidative damage to ECs and delay the re‐endothelialization and finally lead to IH 27, 28.

MSCs can be mobilized after vascular injury, participating in the remodelling processes of the injured vasculature. MSCs exhibited a strong capacity for adhesion to repair vessel wall after injury. On adhesion, a substantial proportion of MSCs differentiated into ECs in the neointima 6, 7, 29. MSCs expressed and secreted VEGF, HGF and other factors. These growth factors and cytokines function together to modulate the local environment, affecting the proliferation, migration, differentiation and functional recovery of resident cells,and leading to early re‐endothelialization and attenuation of IH 30, 31. However, oxidative stress has a deleterious effect on MSC survival and function, thus inhibiting tissue repair 14, 32, 33.

In theory, up‐regulation of the NF‐κB pathway could promote survival of MSCs 15, 34. Other studies suggest that the NF‐κB pathway is involved in regulation of directional migration 35, 36, 37. The NF‐κB pathway widely participates in gene expression and regulation of chemokines, cytokines and adhesion molecules 38, 39. The IκB kinase (IKK) complex consists of two catalytic subunits (IKK‐α and IKK‐β) and a regulatory subunit (IKK‐γ/NEMO). IKK‐α and IKK‐β are critical for NF‐κB to perform its regulatory function 40, 41. In this experiment, we confirm that TNF‐α activates the NF‐κB pathway by increasing phosphorylation of IKK‐α/β.

The percentage of cells in S phase of cell cycle was significantly increased in the TNF‐α group, and the effect can be reversed by IKK XII. We speculate that activation of the NF‐κB pathway could promote cell proliferation. The EdU assay also showed that TNF‐α can significantly enhance the proliferation, supporting the above results. In addition, we have confirmed that the NF‐κB pathway plays the leading role in regulating cell viability by SB203580 antagonizing the p38‐MAPK pathway.

With TNF‐α increasing p‐NF‐κB‐p65, the CXCR4 was up‐regulated as well. Pre‐treatment with IKK XII abolished the overexpression of CXCR4. We considered that TNF‐α promotes CXCR4 expression and enhances migration of MSCs *via* the NF‐κB pathway, consistent with a previous report 42. Furthermore, the transwell migration assay results also support this finding. The cell‐surface phenotype assay demonstrated that the level of CXCR4 expressed on the surface of MSCs increased following treatment with TNF‐α. This may be the direct cause of enhanced migration of ^TNF‐α‐PC^MSCs.

Following treatment with 200 μM H_2_O_2_, the viability of MSCs was markedly decreased, accompanied by a significant increase in apoptosis. Annexin V‐FITC/PI stain indicated that TNF‐α preconditioning reduced the degree of apoptosis, and an increase in anti‐apoptotic protein Bcl‐2 was observed in the TNF‐α group as well. The anti‐apoptotic effect of TNF‐α preconditioning may be related to the up‐regulation of Bcl‐2. TNF‐α also elevated the expression of CXCR4, while oxidative stress down‐regulated it, suggesting that ^TNF‐α‐PC^MSCs would experience increased rates of migration under oxidative stress. These results confirm that TNF‐α reduces apoptosis and improves migration of MSCs under oxidative stress *via* the NF‐κB pathway.

An ELISA test of MDA confirmed the existence of oxidative stress caused by vein grafting. And the TUNEL assay revealed that ^TNF‐α‐PC^MSCs could significantly delay apoptosis of endothelial cells caused by oxidative stress. We speculated that the protective effect of ^TNF‐α‐PC^MSCs on endothelial cells may be related to the secretion of pro‐survival factors, such as VEGF and HGF 43, 44. ELISA results showed that the secretion of VEGF and HGF was significantly elevated in ^TNF‐α‐PC^MSCs, confirming our speculation. Kwon YW applied conditioned medium derived from TNF‐α‐treated MSCs to treat the limb ischaemia and found that IL‐6 and IL‐8 secreted by MSCs could promote homing of endothelial progenitor cells and angiogenesis 45. Paracrine factors of MSCs could be further studied and utilized to alleviate IH or other disease.

The number of ^TNF‐α‐PC^MSCs migrated to the intima of the vein graft was significantly increased, and this increase was halted by IKK XII. In addition, the neointima of the ^TNF‐α‐PC^MSC group was also thinner than ^Non‐PC^MSC group. These data confirm that ^TNF‐α‐PC^MSCs can more efficiently migrate to the damaged vascular intima and better prevent IH in vein graft. The therapeutic effect may come from the synthetic action of enhanced anti‐apoptosis and ameliorative migration of MSCs.

In summary, we have demonstrated that TNF‐α preconditioning can promote survival and migration of MSCs under oxidative stress and promote an increase in number of MSCs migrating to vein graft. As a result, apoptosis of endothelial cells is reduced, and IH of vein graft significantly attenuated. Meanwhile, the NF‐κB pathway participates in the regulation process. Functionally improved MSCs therapy for IH will have good prospects. So far, the clinical trials of stem cell therapy for myocardial infarction or spinal cord injury have obtained the very good results. The breakthrough of the trials indicates the stem cell therapy is striding forward greeting the dawn of the new age.

## Conflict of interest

The authors confirm that there are no conflict of interests.
